# The Emerging and Diverse Roles of Bis(monoacylglycero) Phosphate Lipids in Cellular Physiology and Disease

**DOI:** 10.3390/ijms21218067

**Published:** 2020-10-29

**Authors:** Megan R. Showalter, Anastasia L. Berg, Alexander Nagourney, Hailey Heil, Kermit L. Carraway, Oliver Fiehn

**Affiliations:** 1 NIH West Coast Metabolomics Center, University of California Davis, Davis, CA 95616, USA; mrshowalter2@gmail.com (M.R.S.); ajnagourney@ucdavis.edu (A.N.); hoheil@ucdavis.edu (H.H.); 2 Department of Biochemistry and Molecular Medicine, UC Davis School of Medicine, Sacramento, CA 95817, USA; anastasial.berg@gmail.com (A.L.B.); klcarraway@ucdavis.edu (K.L.C.III)

**Keywords:** bis(monoacylglycero)phosphate, lysobisphophatidic acid, lysosome, lipidomics

## Abstract

Although understudied relative to many phospholipids, accumulating evidence suggests that bis(monoacylglycero)phosphate (BMP) is an important class of regulatory lipid that plays key roles in lysosomal integrity and function. BMPs are rare in most mammalian tissues, comprising only a few percent of total cellular lipid content, but are elevated in cell types such as macrophages that rely heavily on lysosomal function. BMPs are markedly enriched in endosomal and lysosomal vesicles compared to other organelles and membranous structures, and their unique *sn*-1:*sn*-1′ stereoconfiguration may confer stability within the hydrolytic lysosomal environment. BMP-enriched vesicles serve in endosomal-lysosomal trafficking and function as docking structures for the activation of lysosomal hydrolytic enzymes, notably those involved in the catabolic breakdown of sphingolipids. BMP levels are dysregulated in lysosomal storage disorders, phospholipidosis, metabolic diseases, liver and kidney diseases and neurodegenerative disorders. However, whether BMP alteration is a mediator or simply a marker of pathological states is unclear. Likewise, although BMP acyl chain composition may be altered with disease states, the functional significance of specific BMP species remains to be resolved. Newly developed tools for untargeted lipidomic analysis, together with a deeper understanding of enzymes mediating BMP synthesis and degradation, will help shed further light on the functional significance of BMPs in cellular physiology and pathology.

## 1. Introduction

**Figure 1:**
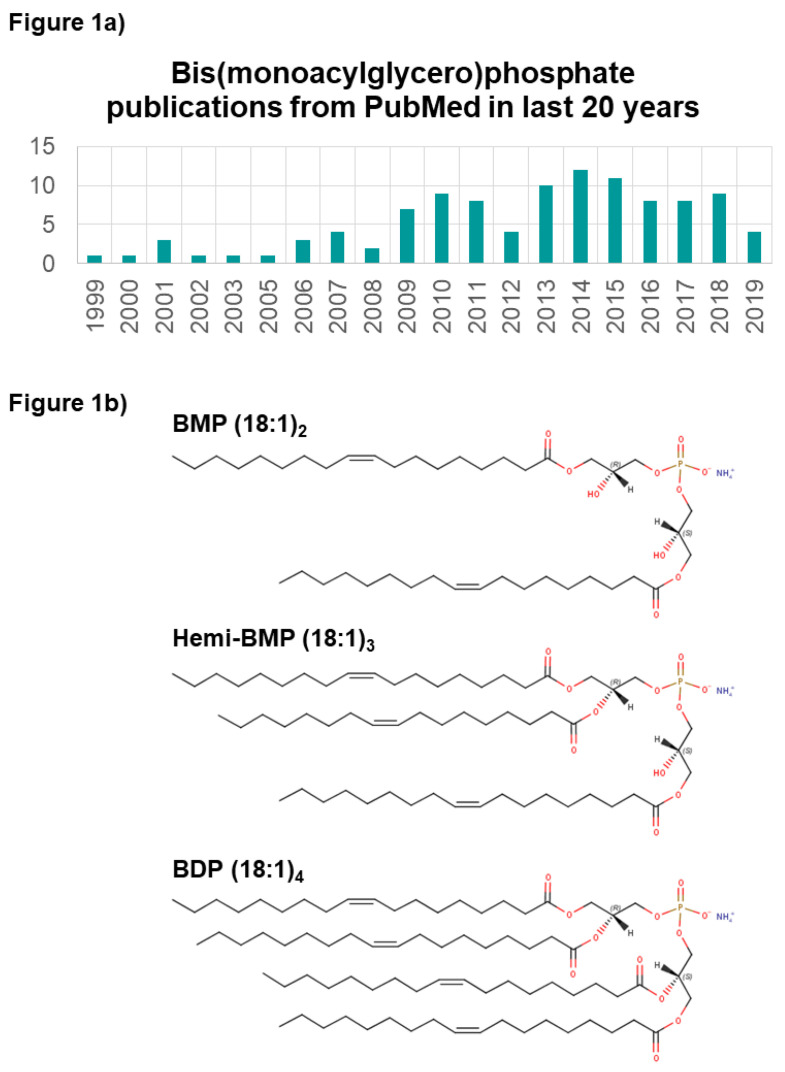
Overview of bis(monoacylglycero)phosphate (BMP) publications and known BMP species. (**a**) Number of publications mentioning BMP in PubMed from 1999 to 2019. (**b**) Known BMP species variants, shown with fatty acyl side chains composed of oleic acid (FA C18:1) as an example.

Recent analytical, cheminformatic, and biological breakthroughs are revealing the diverse roles of lipids in physiology and disease. The function of lipid mediators and other signaling lipids in biological processes have paved the way for the discovery of new lipid classes and characterization of their mechanistic contributions. The phospholipid class bis(monoacylglycero)phosphate (BMP), first identified in 1967, is reportedly key to lysosome stability and vesicle trafficking, and is dysregulated in diseases such as lysosomal storage disorders [1]. The literature has been sparse since the initial discovery of the BMPs, originally named lysobisphophatidic acid (LBPA) [2]. Yet, the last decade has seen a rise in BMP-related publications (Figure 1a), reflecting the increasing recognition of the functional importance of BMPs in membrane biology.

BMPs are negatively charged glycerophospholipids localized almost exclusively within the membranes of late endosomal and lysosomal vesicles [3]. Bis(acylglycero)phosphates may contain multiple acyl chains: two (BMPs), three (known as hemi-BMPs), or four (bis(diacylglycero)phosphates, BDPs); however, very little is known about the function or diversity of hemi-BMPs and BDPs [4,5] in vivo (Figure 1b). In most cells and tissues, BMP is found to comprise less than 1% of total phospholipids [6,7,8,9]. Levels are marginally elevated in some tissue types like rat spleen [10], and very significantly elevated in alveolar macrophages where it represents up to 18% of total cellular phospholipids [6,8,9]. Notably, alveolar macrophage lysosomes are several times larger than average [8], though it is unclear if this vesicle enlargement fully accounts for the increased BMP fraction. BMPs have also been found in human plasma and are thought to be associated with lipoproteins. Plasma BMP levels have been demonstrated to be altered in a variety of diseases or altered in physiological states [1]. Non-mammalian systems have a slightly higher BMP content, as BMP comprises up to 4% of total lipids in bacteria [6]. Studies have shown that BMP may be a relevant biomarker for detecting metastatic cancer of macrophage origin [8] and certain lysosomal storage diseases [1,6], or as a marker for drug-induced phospholipidosis [11,12]. BMP can also elicit strong immunoreactivity against antiphospholipid family antibodies, associated with antiphospholipid syndrome [13]. While the cellular abundance and acyl-chain diversity of BMPs is supported by a growing body of literature, BMP metabolism remains largely uncharacterized.

### 1.1. Mass Spectrometry- and Antibody-Based Measurement of BMPs 

**Figure 2:**
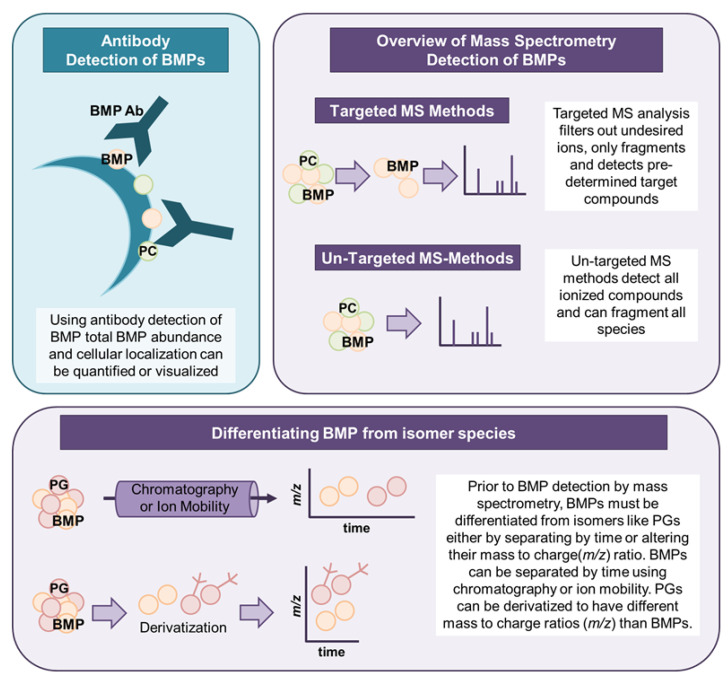
Overview of the detection methods for BMPs. BMPs can be analyzed by antibody detection or by mass spectrometry. Mass spectrometry techniques use targeted or untargeted methods to measure BMPs. BMPs must be differentiated from isomeric phosphatidylglycerol (PG) species during mass spectrometry analysis, which can be accomplished by a number of methods including chromatography or derivatization.

The measurement of BMPs can be performed by either antibody-based or mass spectrometry (MS)-based approaches, as illustrated in Figure 2. The use of commercially available antibodies for BMP detection permits quantification of the entire cellular BMP population but does not allow identification of BMP subspecies with acyl chain or saturation differences. Kobayashi et al. first generated an anti-LBPA/BMP monoclonal antibody (C64) using endosomal membranes isolated from baby hamster kidney (BHK) cells to aid the structural and functional characterization of intraluminal vesicles (ILVs) within late endosomes. BMP localization and enrichment in these internal vesicle membranes was observed using immunofluorescence techniques, and a role for BMP in receptor trafficking within the endosomal system was demonstrated [13]. Antibody-based BMP detection methods have been subsequently applied to study lipid abundance and cellular distribution, and to identify BMP binding partners. Furthermore, cellular internalization of anti-BMP antibody through endocytosis disrupts vesicular dynamics and has been used to probe alterations in late endosomal structure and function [14]. BMPs can also be visualized in live cells using pseudoisocyanine (PIC) dye J-aggregates [15]. However, data obtained by LC-MS or antibodies do not always agree and, hence, antibody results should be interpreted with care.

Depending on the methodologies employed, mass spectrometry (MS) can semi-quantify or quantify BMPs and resolve individual BMP species of varying fatty acyl chain length and saturation. Quantification of BMP species can be achieved by including an isotopically labeled internal standard or by comparison to an external BMP calibration curve. Fatty acyl chain compositions of BMPs are annotated using fragmentation patterns in MS/MS spectra. Collision induces dissociation in tandem mass spectrometry fragments precursor molecules, and the subsequent fragment ions are recorded as the MS/MS (or MS2) spectra. The MS/MS spectra of a compound can be considered a fingerprint pointing to the identity of the compound [16], especially if combined with liquid chromatography retention time information. For identification, experimental MS/MS spectra are then compared to library spectra, which for lipids are largely based upon in silico mass spectral libraries [17,18]. In silico libraries contain putative MS/MS spectra modeled after experimental spectra obtained from a few lipids of the class of interest. Other fatty acyl chain combinations are then modeled using rules established from standard fragmentation, allowing the library to be expanded in the absence of independent analysis of each individual lipid [17,19]. Currently, the only publicly available in silico lipid database for BMP species is from Hiroshi Tsugawa (RIKEN Institute, Japan) and can be downloaded or used in MS-DIAL data processing software [20]. This database includes ESI (+) mode spectra for 1891 BMP species, in addition to 10,206 spectra for hemi-BMPs but does not include diacyl- BMP species.

Recent advances in chromatography and mass spectrometry investigated the separation of BMPs from their phosphatidylglycerol (PG) isomers by other techniques, including ion mobility [21], as highlighted in a specialized review [22]. Isomeric separation is important for mass spectrometry analyses because structural isomers are detected as identical masses and usually cannot be distinguished without additional experimental information such as retention time. Chromatographic separation of BMPs and PGs has been reported using HILIC-MS [23,24,25], RPLC-MS [26,27] or nanoflow LC [28]-based methods to achieve species separation by time for BMPs and PGs. Without chromatographic separation, BMPs can either be differentiated from PGs by fragmentation rules in ESI (+) mode [1] or using derivatization methods to specifically modify BMP species [29]. Derivatization selectively alters the mass of BMPs but not PGs, and allows for analysis without chromatographic separation by shotgun lipidomics [21]. BMPs can also be separated from PGs by ion mobility [30], which utilizes differences in compound mobility in a carrier gas to separate compounds prior to detection. Additionally, thin layer chromatography (TLC) methods can be used to separate BMPs from PGs prior to analysis by either mass spectrometry or staining [31]. The optimal approach to BMP measurement, either antibody- or MS-based, is largely dependent upon the study design and desired data. While there are certainly numerous applications of immunolabeling for functional characterization of cellular BMP, there are clear advantages to using chromatographic and high-resolution mass spectrometry for precise quantitation of BMP abundance and structural classification. The loss of BMP isomer resolution associated with antibody detection in immunohistochemistry is replaced by improved specificity in the quantification of individual BMP species of varying acyl chain length and saturation, and which may have varying biological roles.

### 1.2. Biosynthesis and Metabolism of BMPs in Diverse Tissues

The metabolism of BMPs has not been fully elucidated, and there are competing theories regarding the biosynthesis and catabolism of BMP [7]. BMP is reportedly synthesized from phosphatidylglycerol [32], and while mechanisms have been proposed for the conversion of PG to BMP [7], uncertainty persists regarding formation of BMP’s unique stereochemistry [6,7]. All other mammalian glycerophospholipids have *sn*-3 glycerophosphate stereo configuration, while BMPs can reportedly possess *sn*-1:*sn*-1′ as well as *sn*-1:*sn*-3’ and *sn*-2:*sn*-2’ stereoconfiguration [6,33]. Many have argued that BMP’s unusual stereo configuration and negative charge at lysosomal pH protects it from degradation by lysosomal enzymes [6] As suggested by Hullin-Matsuda et al., it is possible that the application of modern methods to understand BMP stereoconfiguration will be necessary for a full understanding of its metabolism [6].

Manipulating cellular BMP concentrations has been shown to regulate endo-lysosomal lipid metabolism and membrane dynamics [7]. ABHD6 is a BMP hydrolase responsible for up to 90% of BMP catabolism in vivo, and co-localizes with lysosomal and late endosomal membranes but exhibits a pH optimum between 7.5 and 8.0, lacking enzyme activity at acidic pH [34]. TLC analysis suggests that ABHD6-mediated hydrolysis of BMP results in the accumulation of lysophosphatidylglycerol and free fatty acids [34]. ABHD6 apparently does hydrolyze PG and degrades BMP species independent of stereochemistry [34]. ABHD6 was also shown to degrade 2-arachidonoylglycerol (2-AG) in the brain [35], and was found to function as both a monoacylglycerol lipase and a lysophospholipase [36]. However, ABHD6 substrate preferences in this study were based on changes in the abundance of lipid species after treatment with antisense oligonucleotide targeting ABHD6 [36], and may include effects of downstream enzymes.

Inhibition of ABHD6 increases total BMP levels as well as long chain and fully saturated BMP species in mouse livers. When animals were fed high fat chow, livers exhibited elevated BMP levels and enrichment of long chain BMP species relative to control animals [34]. Knockdown of ABHD6 was found to protect mice from diet-induced obesity [36], but values for BMP species were not reported. Inhibition of ABHD6 with a small molecule (WWL70) reduced inflammatory processes in a mouse model of acute lung injury by reducing neutrophil infiltration, cytokine secretion, and protein extravasation, but this study did not measure changes in lipid species other than 2-AG [37] and might have benefitted from untargeted lipidomics methods. Deletion of ABHD6 increases circulating BMP levels in mouse plasma, and BMP was found to accumulate in HDL fractions [38]. In summary, it is clear that ABHD6 mediates BMP abundance, and altering ABHD6 function results in pronounced phenotypic effects, particularly under stress.

Three other enzymes have reported hydrolase activity toward BMP species. PA-PLA2/MGL, a member of the carboxylesterase family, has been shown to hydrolyze BMP, assessed by the production of free fatty acids in BMP-rich lipid fractions prepared from liver lysosomes [4]. Pancreatic lipase related protein 2 (PLRP2), with phospholipase A1 activity, can also hydrolyze BMP and hemi-BMP [4]. Finally, ABHD12 exhibits weak hydrolytic activity toward BMP species but is predominately a lysophosphatidylserine lipase [39].

### 1.3. BMP Esterified Acyl Chain Composition and Changes

The composition of BMP fatty acyl chains is thought to contribute to the biochemical functions of the various BMP species [7,8]. In numerous tissues and cell types, oleic acid (FA 18:1) was found to be the major fatty acyl constituent [21,24,40,41,42,43]. The polyunsaturated fatty acids linoleic acid (FA 18:2), arachidonic acid (20:4), and docosahexaenoic acid (22:6) have also been identified in significant concentrations in various cell types [6]. Alterations in fatty acid composition have been associated with diet [21] and drug treatment [24], further supporting the suggestion that BMP’s fatty acyl composition is dynamically regulated, though mechanistic insight is lacking. In vitro studies have shown that BMP levels increase as a result of both esterified fatty acid (supplemented as PGs) and non-esterified fatty acid supplementation [44,45], highlighting that BMP fatty acyl composition can be manipulated by changing fatty acyl or PG pools. 

Improved analytical methods have prompted the more widespread investigation of BMP acyl chain composition, and several studies have reported varying compositions of BMP in response to cellular stress or pathological conditions. Bouvier et al. demonstrated that fatty acyl chain composition dictates the stability of BMP species under oxidative stress. Supplementation with either PG-18:1/18:1 or PG-22:6/22:6 remodeled BMP acyl chain composition accordingly, and altered BMP sensitivity to oxidative stress-induced degradation in macrophages [44]. While the authors observed BMP degradation, specific degradation products were not examined. The incomplete knowledge of BMP biosynthesis and catabolism and the unknown roles of fatty acyl remodeling enzymes in these processes highlight the need for additional studies to elucidate the metabolism of individual BMP species.

BMP abundance and side chain composition also vary with the onset of lysosomal storage disease in patient plasma and cultured fibroblasts [1]. Numerous lysosomal storage disease (LSD) fibroblasts were assayed; Gaucher, Mucopolysaccharidosis type I (MSPI), MPSII, MPSIIIA, Niemann-Pick Disease Type A/B/C and Pompe cells all showed different BMP species distribution relative to control cells. Oddly, BMP profiles in Fabry disease cells were nearly identical to those in control cells. Niemann-Pick Disease Type C cells contained almost no long chain BMP species and strongly accumulated BMP 18:1_18:1 compared to control cells. Elevated BMP 18:1_18:1 and reduced long chain BMP species were also reported in other LSD cell types [1]. PGs abundance was not significantly altered in LSD cells compared to control cells, with the exception of Fabry disease [1], indicating that alterations in BMP content are not due to decreased substrate abundance but rather altered BMP metabolism.

In an in vivo model of Gaucher disease, fatty acid supplementation increased total BMP species [45]. The authors only reported data from analyses of diseased cell and not control cells, so it is unclear if the observed BMP changes in Gaucher disease macrophages indicate a disease-specific phenotype. Interestingly, the authors chose to supplement oleic (FA 18:1) or linoleic acid (FA 18:2), which are two closely related fatty acid species, so alterations in BMP levels could be attributed directly to fatty acid chain saturation, as chain length remained constant. Oleic acid (FA 18:1) supplementation dramatically increased the abundance of not only BMP esterified species containing oleic acid but also all other BMP species, including long chain BMPs [45]. This is an interesting result, as linoleic acid is an essential fatty acid, indicating that the reduction in BMP was not due to decreased availability of long chain BMP fatty acid precursors but instead to deliberate incorporation of fatty acid groups in BMPs in the Gaucher disease state by an unknown mechanism. 

### 1.4. BMP is a Structural Lipid Important for Lysosomal Protein Membrane Docking and Function 

BMP plays a critical but historically underappreciated role in lysosome biology, as recent reports implicate BMP in the regulation of lysosomal stability, function, enzyme activation, and endosomal trafficking. BMP’s negative charge is retained within the acidic environment of the lysosome, allowing it to act as a docking site and essential cofactor [46] for some lysosomal proteins that contain positively charged domains [3,46,47]. Proteins known to interact with BMP include hydrolases such as acid sphingomyelinase (ASM), lysosomal phospholipase A2 (LPLA2) and acid beta-glucosidase, as well as heat shock proteins such as Hsp70, saposin activating proteins (SAPs), and apoptosis linked gene 2 interacting protein X (Alix), among others [6,7,48,49]. BMPs are also known to play an important role in the endocytic pathway [6], affecting membrane curvature, protein cofactor recruitment, and endosomal trafficking. Their structural and chemical properties can modulate membrane invaginations in BMP rich domains [6]. In coordination with the ESCRT and Alix proteins, BMPs are involved in both the creation of ILVs and the fusion of ILVs with the limiting endosomal membrane [50].

## 2. Acid Sphingomyelinase

**Figure 3:**
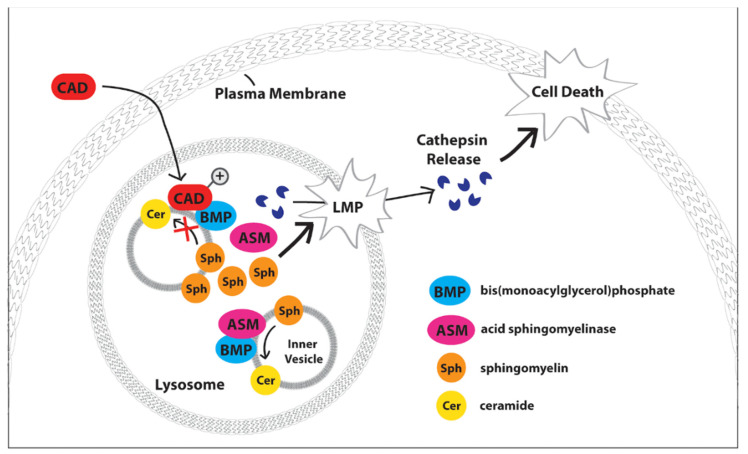
Schematic of BMP’s functional role within lysosomes. BMP serves as a docking site and critical cofactor for acid sphingomyelinase (ASM), which metabolizes sphingomyelin (Sph) to ceramide (Cer) at intraluminal vesicles (inner vesicles). Cationic amphiphilic drugs (CADs) rapidly partition across cellular membranes and become protonated and sequestered within the acidic lysosomal lumen in a process termed ‘lysosomal trapping.’ Within the lysosome, CADs disrupt acid ASM-BMP interactions, thereby inhibiting ASM function and causing buildup of sphingomyelins. Altered sphingomyelin metabolism destabilizes the lysosomal membrane, which can lead to lysosomal membrane permeabilization (LMP), consequent lysosomal cathepsin protease release, and cell death.

ASM is one of three types of sphingomyelinase hydrolases that cleave sphingomyelins into ceramides, and is primarily localized in the lysosome [51], though it is also found within the extracellular leaflet of the plasma membrane [52]. ASM has been implicated in the hydrolysis of sphingomyelins in both the endo-lysosomal compartment and the outer plasma membrane leaflet. Encoded for by SMPD1 (sphingomyelin phosphodiesterase 1), alternative post-translational modifications target ASM to either the plasma membrane or the endo-lysosomal compartment [53]. At lysosomal pH, ASM activity is highly stimulated by anionic lipids such as BMP [54], and ASM has been found to contribute to lysosomal membrane stability through interactions with heat shock protein 70 (Hsp70) [47]. ASM binds preferentially to BMP within the lysosome [48], and disruption of this interaction by lysosomotropic agents including cationic amphiphilic drugs inhibits ASM function, leading to altered sphingolipid metabolism (Figure 3) [3]. Disruption of BMP-dependent conversion of sphingomyelin to ceramide by ASM [46] has been indirectly but crucially connected to lysosomal lipid extraction and degradation important to maintaining lysosomal stability [7]. 

### 2.1. Niemann-Pick Disease Type C2 Protein (NPC-2) 

The cholesterol transfer protein NPC-2 shuttles cholesterol from intralysosomal membranes to the NPC-1 protein in the perimeter/limiting lysosomal membrane [7,48]. NPC-2 cholesterol transfer activity is stimulated by ceramide and inhibited by sphingomyelin [7,50]. Enkavi et al. reported that BMP is required for NPC-2 binding in prone mode (i.e., the cholesterol binding pocket is in contact with the membrane), while sphingomyelin inhibits BMP-NPC-2 binding in prone mode but not supine mode (cholesterol binding pocket is facing away from the membrane) [55]. From these studies, it is clear that BMP binding to NPC-2 is important for cholesterol transport within the lysosome, emphasizing how alterations to BMP’s enzyme binding and cofactor function can affect lysosome biology and produce a range of phenotypic changes depending upon local protein and lipid levels.

### 2.2. Heat Shock Protein 70 (Hsp 70)

Lysosomal Hsp70 binds BMP with high affinity and specificity and is known to stabilize lysosomal membranes, protecting against lysosomal membrane permeabilization (LMP) [47]. The extent of membrane permeabilization or rupture depends on the stimulus, but LMP is broadly characterized by the leakage of lysosomal content into the cytosol, often leading to lysosome-mediated cell death [56]. Hsp70 facilitates BMP binding to ASM and consequent regulation of lysosomal sphingomyelin metabolism, which is critical to lysosome stability and LMP prevention. Lysosome stability induced by Hsp70 overexpression was reversed by the inhibition of Hsp70-BMP interaction through BMP-directed antibodies and an Hsp70 point mutation, as well as by pharmacological or genetic inhibition of ASM [47]. It has been proposed that increased lysosomal ceramide production resulting from enhanced ASM activity causes enhanced organelle fusion with other intracellular vesicles or the plasma membrane [47], which further serves to stabilize the lysosome by altering its volume and lipid composition [47]. Further investigation into BMP’s larger role in LMP prevention aside from the Hsp70-dependent mechanism described here is warranted.

### 2.3. Lysosomal Phospholipase A2 (LPLA2)

The phospholipase A2 family represents a diverse group of over 15 enzymes that can be classified into subgroups based on cellular location [57]. Group XV PLA2, or lysosomal phospholipase A2, is an enzyme responsible for the catabolism of endogenous phospholipids and plays a central role in cellular lipid homeostasis. Dysregulation of LPLA2 can lead to phospholipidosis [58,59], the general accumulation of lipids in vesicular membranes often arising from altered lipid metabolism. Abe and Shayman found that BMP was the strongest stimulator of LPLA2 among negatively charged lipid species tested in liposomes [58]. BMP’s unique conformation, negative charge, and resistance to degradation are thought to be pivotal to its docking and activation of LPLA2 [58,60].

### 2.4. Apoptosis Linked Gene 2 Interacting Protein X (Alix)

While Alix reportedly binds BMP, the functional significance of this interaction is debated. Alix is essential in the formation intra-lysosomal vesicles, and it has been proposed that its interaction with BMP at the limiting lysosomal membrane facilitates this process [6,7,48,49]. However, a recent study found that the perimeter lysosomal membrane is virtually devoid of measurable BMP [7], raising questions regarding this hypothesis [7]. Alix and BMP have each independently been shown to regulate cholesterols levels [6,7,48,49], but the mechanism remains unknown [6].

## 3. BMPs and Disease

BMPs are tied to a broad range of diseases, with direct links to mechanisms of disease initiation or progression, or as markers of disease status. As noted above, BMPs can provide scaffolding to facilitate endosomal trafficking and lysosomal membrane maintenance through association with target proteins. Increased BMP levels have been linked to several well-studied lysosomal lipid storage diseases including NPC, GM1 Gangliosidosis [3], and Gaucher disease [45]. Data from primary cell culture studies report varied associations between BMP abundance and acyl chain group enrichment across disease subtypes [1]. BMPs are also implicated in acquired lysosomal storage disorders (often induced from cellular stressors such as cationic amphiphilic drugs), as well as antiphospholipid syndrome and Stargardt disease [61,62]. 

Biomarkers for lysosomal storage diseases [1] and a variety of other physiological and cellular conditions, including inflammation in late resolving state [63], lipid storage disorders in macrophage and liver cells [6], and drug-induced phospholipidosis [11], among others. Table 1 summarizes disease-associated proteins known to interact with or to be affected by BMPs, along with disease occurrence rates and specific BMP species alterations, if known. With improvements in the measurement of BMPs, more studies are reporting enrichment in various BMP species. From the reported enrichments in a range of tissues, clear patterns are emerging with respect to consistently enriched side chains.

### 3.1. Lysosomal Storage Diseases Display Unique BMP Associated Phenotype

Lysosomal storage diseases (LSDs) are a group of inherited metabolic disorders caused by genetic defects in lysosome resident proteins [64,68], characterized by the lysosomal accumulation of enzymatic substrates [64,68]. Incidence of individual LSD subtypes are rare but as a group occur in approximately one in every 7700 live births [64]. LSDs are grouped into four broad classes based on their substrate type: mucopolysaccharidoses, lipidoses, glycogenoses, and oligosaccharidoses [64]. Disorders in enzymes leading to the accumulation of compounds within each class, as well as across compound classes, share many phenotypic and clinical similarities [64]. Common clinical features include bone abnormalities, organomegaly and central nervous system dysfunction [64]. In most lysosomal lipid storage diseases (lipidoses), these phenotypic features arise from the dysfunction of a specific lysosomal lipid metabolizing enzyme, leading to the accumulation of the substrate, and the precipitation of other hydrophobic substrates within the endolysosomal system, resulting in a membrane transport “traffic jam” [3]. Disruption of lysosomal function then leads to cellular starvation through impairment of nutrient delivery from the endolysosomal system [3]. However, many specific mechanisms underlying lipidosis disease progression and LSD pathogenesis are currently incompletely understood [68]. Therapeutic approaches are limited [3], but therapies targeting BMP hold future promise [45,47,49,69].

#### 3.1.1. GM1 and GM2 Gangliosidosis

Gangliosides are the main glycolipids of neuronal plasma membranes [70], and both GM1 and GM2 gangliosidoses are fatal neurodegenerative diseases [70]. Importantly, the hydrolysis of both GM1 and GM2 gangliosides is promoted by BMP [70]. In non-affected humans, BMP-stimulated GM1 β-galactosidase cleavage of terminal β-D-galactose from ganglioside GM1 produces GM2 [3]. GM2 is hydrolyzed by cooperation between the GM2 activator protein and the β-hexosaminidase protein [70]. An inherited deficiency of the lysosomal GM1 β-galactosidase results in GM1 gangliosidosis, characterized by progressive neurological symptoms tied to accumulation of lipid species [3]. Inherited deficiencies in either the GM2 activator protein or the β-hexosaminidase result in GM2 gangliosidosis [70].

#### 3.1.2. Gaucher Disease

Gaucher disease is caused by an inherited deficiency in acid β-glucosidase, resulting in impaired degradation of glucosylceramide (GC) [3,45]. Under normal conditions, acid β-glucosidase cleaves GC to produce glucose and ceramide, but impaired metabolism causes the accumulation of GC, as well as dihexosylceramide (DHC) and trihexosylceramide (THC) in Gaucher disease [45]. Deficiencies in the protein prosaposin and its cleaved active forms (saposins Sap-A, Sap-B and Sap-C) can also lead to an abnormal form of Gaucher disease with a corresponding accumulation of GC [3,48]. Sap-C allosterically binds to and activates acid β-glucosidase to promote the lysosomal degradation of GC [48]. BMP has been shown to strongly stimulate GC hydrolysis [71], likely by acting as a negatively charged ILV docking site for the Sap-C/acid β-glucosidase complex. At lysosomal pH, Sap-B is a positively charged, glycosylated lipid binding protein and is structurally similar to Sap-C [72]. Sap-B purportedly aids lysosomal degradation by binding to negatively charged phospholipids, primarily BMP, within intralysosomal membranes and aiding transfer of substrate lipids for degradation [72,73]. BMP’s crucial role in this process reflects its function as a negatively charged surface for Sap-B binding to result in membrane perturbation [73], and sap-B function is enhanced with increasing BMP concentration in vitro [72].

In a macrophage cell line model of Gaucher THP-1 disease in which acid beta-glucosidase was inhibited, BMP levels increased in disease cells compared to control. The increase in BMPs was suggested by the authors to occur mainly as a consequence of lysosomal expansion-associated GC accumulation [45]. Total BMP concentration decreased in this model when oleic (FA 18:1) or linoleic acid (FA 18:2), capable of integrating into BMP, was added to the culture media [45]. For reasons mechanistically unknown, this inclusion of linoleic acid and the subsequent decrease in total BMP concentration was accompanied by a reduction in GC, DHC, and THC, but only when cells were supplemented prior to GC accumulation [45]. Based on these observations, the authors suggest a role for BMP in regulating lysosomal storage capacities [45], though this has yet to be mechanistically characterized.

#### 3.1.3. INCL, MPS1, MPS2 and Fabry Disease

Elevated BMP levels have been identified in brain tissue from patients with infantile neuronal ceroid lipofuscinoses (INCL) [6,74] as well as in cultured skin fibroblasts derived from patients with MPS1, MPS2 and Fabry disease [1,6]. All four of these lysosomal storage diseases result from defects in their respective enzymes [1,3,6,74], but their direct connection to BMP remains inconclusive [6].

#### 3.1.4. Niemann-Pick Disease Type C (NPC)

Mutations in either the NPC-1 or NPC-2 genes lead to Niemann-Pick C (NPC), also characterized by abnormal lipid storage and accumulation [3]. As mentioned previously, the cholesterol transfer protein NPC-2 transfers cholesterol from intra-lysosomal membranes to the NPC-1 protein in the perimeter lysosomal membrane [7,48]. The NPC family of diseases clinically present heterogeneously; symptoms range from neurological to psychiatric and present across a range of patient ages [75]. At the cellular level, NPC is characterized by accumulations of myeloid bodies, also known as multi-lamellar bodies. Di-22:6 BMP accumulates in urine of Niemann-Pick Type C patients and is considered a disease biomarker [11].

### 3.2. BMP Modulation in Other Genetic or Acquired Disorders

#### 3.2.1. Stargardt Disease

Stargardt disease is an inherited form of macular degeneration characterized by juvenile-onset progressive vision loss, and is most commonly associated with defects in the ATP-binding cassette gene sub family A-4 transporter protein (ABCA4, or ABCR) [61]. ABCA4 protein loss or dysfunction results in buildup of lipofuscin, a major component of which is the cationic amphiphile N-retinylidene-N-retinylethanolamine (A2E) [61]. Accumulation of A2E has been observed in both knockout ABCA4 mouse models and Stargardt patients [61]. In vitro, the accumulation of A2E is protective, inhibiting lysosomal degradation in photoreceptors. It has been hypothesized that A2E’s accumulation plays a major role in the pathogenesis of Stargardt disease and that similar mechanisms may occur in age-related macular degeneration [61]. 

It has been suggested that BMP may play a role in the progression of Stargardt disease [61], though clear mechanistic studies are lacking. BMP, like A2E, accumulates in ABCA4 knockout model lysosomes and in patient retinal tissue. As A2E is a cationic amphiphile, BMP accumulation likely occurs in a mechanistically similar fashion to BMP accumulation following exposure to cationic amphiphilic drugs [61] (see below). BMP accumulation is considered critical for disease progression, as docosahexaenoic acid (DHA; FA 22:6) is a major fatty acyl constituent of BMP, and DHA is critical for photoreceptor cell recycling and survival [61]. It has been suggested that DHA sequestration by BMP prevents DHA from participating in photoreceptor cell survival and thus may contribute to the pathology of the disease [61]. While this is an interesting mechanism by which BMPs could contribute to disease progression, it has not been clearly demonstrated that DHA levels are limiting in this model, and the topic warrants further study.

#### 3.2.2. Antiphospholipid Syndrome (APS)

Unlike most of the previously described diseases, antiphospholipid syndrome is an autoimmune disease arising primarily from antiphospholipid antibody binding to beta2-glycoprotein1 (B2GP1) [6,76], which leads to increased risk of venous, arterial, and microvascular thrombosis [6,76]. Antibodies from APS patient sera are able to bind BMP [13], and when endocytosed by BHK fibroblasts [13] and human endothelial cells [77] can affect the multifunctional insulin like growth factor/mannose-6-phosphate receptor (IGF2/MPR) [6,13,77], suggesting that BMP may be involved in APS pathogenicity [6]. 

#### 3.2.3. Phospholipidosis, Cationic Amphiphilic Drugs, and Acquired Lysosomal Storage Diseases

Cationic amphiphilic drugs (CADs) are compounds that carry a neutral charge in the cytoplasm but are protonated and positively charged upon entering the lysosome, consequently becoming trapped and concentrated within the organelle [78,79]. Previous studies have shown that CAD administration to cells can induce a phospholipidosis phenotype similar to that of NPC disease wherein sphingomyelin, BMP, cholesterol, and other lipids accumulate in the lysosome, depending on the specific CAD’s mechanism [79]. It is thought that CADs achieve this phenotype by inhibiting lysosomal lipid metabolism, which leads to lipid accumulation and ultimately to the expansion of the lysosomal compartment [79]. Funk et al., reported a four-fold increase in lysosomal volume following exposure of human fibroblasts to imipramine secondary to lipid accumulation, supporting this hypothesis [79].

Comparable to the acquired lysosomal storage disease state triggered by CAD activity, multilamellar and myeloid body formation has been observed in kidney tissue in response to high fat diet, linking this metabolic syndrome with increased risk of kidney disease [80]. Mice that were fed high fat diets developed renal dysfunction and exhibited accumulation of BMP species and other lipids [80]. Specifically, a two-fold increase in levels of Di-22:6 BMP was observed in human urine samples from 21 patients with metabolic and kidney disease [80]. The authors also observed signs of cellular damage, inflammation, and fibrotic and lipogenic marker elevation, transcriptional changes in mice and humans with metabolic disease [80]. The observations provide a new understanding of metabolic disease-associated changes in kidney function, and implicate BMP as a novel biomarker for early detection of disease progression [80].

While alterations in BMP metabolism clearly correlate with the induction of metabolic disease states including CAD-induced phospholipidosis/acquired LSD and other non-LSD diseases, BMP function in the pathophysiology of these conditions is poorly understood. It was recently reported that BMP fatty acyl side chain composition differed between diet- and drug-induced phospholipidosis and in various human disease states, and that deletion of ABHD6 increased circulating BMP levels, effectively reverting the pathological phenotype [38]. Signature BMP abundance profiles and acyl-chain patterns were observed in murine liver and plasma in response to several phospholipidosis inducers including the CAD amiodarone as well as a high fat diet chow and western-type diet chow. The greatest increase in BMP abundance was observed in response to high fat diet, and animals on this dietary regimen displayed specific enrichment of BMP species 36:2, 40:7 and 44:12. CAD-induced BMP alterations in the blood plasma were less pronounced than in the liver. BMP 44:12, a marker for drug-induced phospholipidosis [11,12], was also found with a high fat diet [38]. These findings have implications for management of metabolic diseases, non-alcoholic fatty liver disease (NASH), and liver cirrhosis. Subsequent findings from Grabner et al. found BMP concentrations adapted to changes in ambient temperature and nutritional state of mice in a tissue-specific manner [81]. Further tying involves changes to BMP levels and alterations in the metabolic state. 

In mice, deletion of ABHD6 increased circulating BMP levels, but a corresponding accumulation of hepatic BMP and multilamellar body formation indicative of acquired LSDs was not observed [38]. This implies that BMP alone does not trigger disease progression in hepatic tissues, but that elevation of BMP in response to LSD onset may be compensatory. To understand if BMP could be used to monitor liver disease progression, the authors measured BMP levels in plasma of patients with non-alcoholic fatty liver disease (NAFLD), non-alcoholic steato-hepatitis (NASH), non-alcoholic liver cirrhosis (NALC) and alcoholic liver cirrhosis (ALC). Total BMP levels most dramatically increased in ALC, and secondarily in NALC. Thus, total BMP content alone would not be a robust marker for non-alcoholic liver diseases, but could inform cirrhosis progression. Individual BMP species were more specifically altered in NAFL and NASH, with the ratio of BMP 36:2/(BMP 36:3 + BMP 36:4) significantly increased in both NAFL and NASH relative to control [38]. From this study, the differences observed in individual BMP species in a range of liver diseases and conditions highlight an as yet not understood role for BMPs in liver disease progression and monitoring.

#### 3.2.4. Infection and Inflammation

Considering the critical role BMP plays in endosomal trafficking, including the fusion of late endosomal membranes, it is unsurprising that BMPs have been studied in the context of infection and immune cell function. The BMP-Alix interaction has been reported to contribute to viral invasion. BMP localized to the cytosolic leaflet of late endosomes recruits Alix and its associated proteins [50]. Once bound to BMP, Alix undergoes a conformational change that perturbs membrane symmetry, creating an invagination that allows fusion of ILVs from the luminal side or formation of a new ILV [50]. The ILV pathway is hijacked by certain viruses including the vesicular stomatitis virus (VSV), lassa virus, and lymphocytic choriomeningitis virus, extorting existing cellular machinery to gain entrance to the host [50]. Whether the mechanism of BMP-mediated viral invasion may be exploited to treat or prevent viral infection remains to be explored. Other diseases linked to BMP dysregulation, including CAD-induced phospholipidosis resembling NPC disease, have been shown to inhibit Ebola virus infection [82]. Furthermore, epidemiological studies indicate that NPC patients are resistant to the Ebola virus [82]. This suggests that CADs such as amiodarone or dronedarone that induce this phenotype could provide resistance to the Ebola virus [82]. Early reports in the study of the antiviral effects of chloroquine indicate that BMPs could play a role in the mechanism of protection against enveloped viruses such as SARS-COV-2 [83].

The antiviral mechanism of these CADs may be explained by the characteristic lysosomal lipid accumulation induced by drug administration, which disrupts the endocytic pathway [82]. As BMP is required for proper function of the endocytic pathway, it is not surprising that BMPs are required for membrane fusion of the VSV and dengue viruses [84]. Additionally, modulation of ABHD6 activity has been found to alter immune response in a murine model of lung inflammation, though BMP’s role in promoting a favorable immune response is unclear [37]. In response to ABHD6 inhibitor WWL70, 2-arachidonoylglycerol (2-AG) and lysophospholipid levels increased, as measured by mass spectrometry. Unfortunately, the authors did not directly investigate BMP levels following WWL70 administration, though it is probable that small molecule-based ABHD6 inhibition resulted in increased BMP levels analogously to previous reports in which ABHD6 was mutationally inactivated [34]. However, inhibition of ABHD6 did clearly reduce inflammation, and further studies should investigate whether BMP is directly involved in the production of favorable immune responses.

#### 3.2.5. Age Related Neurological Disease Progression

Alterations in endosomal/lysosomal function are key etiologies of aging-associated neurological diseases including Alzheimer’s and Parkinson’s diseases. The involvement of autophagy and lysosomal function in the progression of Alzheimer’s disease and Parkinson’s disease, amongst others, has been reviewed previously [85]. For example, BMP-binding to Heat-shock protein70.1 (Hsp70.1) is important as a lysosomal stabilizer, and has been implicated to neuronal death in Alzheimer’s disease [86]. BMPs also directly interact with acid sphingomyelinase and increase the hydrolysis of sphingomyelins to ceramides [86]. A common theme in the progression of these neurological disorders is the accumulation of substrates typically degraded and recycled during the autophagic process, including lipids. As cellular degradation is inhibited, it has been reported that cells compensate by excreting contents in exosomes. Miranda et al. reported that disruption of phosphatidylinositol 3-kinase vacuolar sorting protein (Vps34) activity and phosphatidylinositol-3-phosphate (PIP3) levels resulted in autophagic and lysosomal disruption in neural tissues, promoting the excretion of exosomes with unique lipid signatures. The signature included significantly increased concentrations of BMPs [87], both in terms of total lipid abundance and sub-species enrichment. The authors argue that BMPs are not regularly found in exosomes and are therefore only enriched in response to stress due to inhibition to endosomal dysfunction. Reports of BMP content in exosomes have been previously reviewed and further support the lack of enrichment of BMPs in exosomes under most cellular conditions [88]. As BMPs are reported biomarkers in plasma and urine for numerous physiologic conditions [89], it is interesting to consider whether exosomes excreted in these disease states would also exhibit similar changes in BMP enrichment. These investigators found exosomes enriched with amyloid precursor protein c-terminal fragments (APP-CTFs) and BMPs to be biomarkers for the endosomal dysfunction of neurodegenerative disorders, especially those associated with aging.

## 4. Future Directions

Although our understanding of BMPs has unfolded slowly since their discovery over 50 years ago, emerging cellular and clinical observations are beginning to shed light on the contributions of this unique lipid class to tissue homeostasis and pathology. At the cellular level, BMPs are highly localized to late endosomes and lysosomes and appear to play key roles in maintaining lysosomal structural integrity by facilitating cholesterol redistribution and glycolipid trafficking and degradation. Their enriched presence within intraluminal vesicles serves as activating docking sites for several lysosomal enzymes and accessory proteins involved in glycolipid breakdown. Clinically, elevated BMP levels, or alterations in BMP-linked acyl chains, are associated with a variety of pathological states. For example, elevated BMP is a feature of the lipidosis class of lysosomal storage diseases, where the function of key glycolipid degradative enzymes is disrupted but is also observed as a response to treatment with cationic amphiphilic drugs. Considering the variety of states that exhibit BMP dysregulation, an interesting question concerns whether alterations in BMPs might be associated with other diseases such as cancer, where biomarkers for aggressive disease are sorely needed. Moreover, the extent to which BMPs play causative, compensatory or passive roles in each of the pathological states where their levels or acyl chain content are altered defines a key set of questions for future studies.

A second series of questions concern the mechanisms underlying BMP accumulation and acyl chain modification in the pathological state. For example, why is the lipid activator of a compromised lysosomal enzyme elevated in LSD when enzyme activation is biochemically upstream of the genetic lesion? Moreover, why do BMP levels respond to CADs when BMP is upstream of the drugs’ presumed targets? While it has been suggested that elevated BMP is simply the outcome of elevated lysosomal content in cells storing glycolipids, the possibility remains to be explored that the lysosomal accumulation of some glycolipid substrates triggers an adaptive mechanism to bolster BMP levels in an effort to promote the degradation of these species. Exploration of the notion that BMP levels may be tunable and regulated in response to environmental conditions or lysosomal substrate load will require a deeper understanding of the enzymes and metabolic pathways contributing BMP synthesis and degradation. Likewise, the unexplored possibility that alterations in the acyl chain incorporation into BMP contribute to its efficacy as a lysosomal enzyme activator or to its own metabolic stability will require a deeper understanding of the metabolic pathways governing BMP esterification.

## Figures and Tables

**Table 1:** BMP-associated diseases and proteins implicated in disease progression.

Protein	Disease Implicated in	Disease Prevalence (Births)	BMP Levels, Side Chain Composition and Tissue Localization
ASM (Acid sphingomyelinase)	NPA and NPB (Niemann Pick A and B) [3] and Cancer [49]	1–248,000 [64]	Significantly elevated in spleen and liver [65], as well as plasma [1] in patients with NPA and NPB. Di-18:1 BMP and di-18:2 BMP were the predominant side chains in plasma [1].
Acid-beta-glucosidase	Gaucher disease [45]	1–57,000 [64]	Di-18:1 BMP is significantly elevated in plasma samples from patients with Gaucher disease [1].
NPC-1 and NPC-2	NPC (Niemann Pick C) [3]	1–211,000 [64]	Significantly elevated in spleen and liver [65], as well as plasma [1] in patients with NPC. BMP di-18:1 was the most prevalent side chain in plasma [1].
GM1-B-Galactosidase	GM1 gangliosidosis [3]	1–384,000 [64]	Significantly elevated in human brain samples from postmortem patients with GM1 gangliosidosis [9]. BMP di-22:6, di-18:0 and di-18:1 were the most abundant species [9].
Arylsulfatase A	Metachromatic Leukodystrophy [3]	1–92,000 [64]	No significant elevation in plasma [1] but significant elevation in urine observed, however, PG and BMP were undifferentiated in the study’s Mass Spectrometry analysis, so BMP confirmation is unclear [66]
Alpha-galactosidase A	Fabry disease [3]	1–117,000 [64]	Significantly elevated in cultured skin fibroblasts from patients with Fabry disease, with di-22:6 BMP then di-18:1 BMPs as the most abundant species [1].
B2GP1 (Beta-2-glycoprotein-1)	APS (Antiphospholipid syndrome) [6]	1–2000 [67]	NA
IGF2/MPR (insulin like growth factor/mannose-6-phosphate receptor)	APS (Antiphospholipid syndrome) [6]	1–2000 [67]	NA
ABCA4 (ATP-binding cassette, sub family A, member 4)	Stargardt [61]	1–9000 [62]	Di-22:6 BMP and BMPs with C20:4 side chains were significantly elevated in human retina tissues from patients with Stargardt disease [63].
